# Non-specific complaints in the ambulance; predisposing structural factors

**DOI:** 10.1186/s12873-015-0034-5

**Published:** 2015-05-15

**Authors:** Maaret Castrén, Lisa Kurland, Sofia Liljegard, Therese Djärv

**Affiliations:** Department of Clinical Science and Education at Södersjukhuset, Karolinska Institutet, Stockholm, Sweden; Section of Emergency Medicine, Södersjukhuset, Stockholm, Sweden; Emergency Department, Karolinska University Hospital, Solna, Sweden

**Keywords:** Aged, EMS, EMCC, Dispatch, Decision support techniques, Diagnostic errors

## Abstract

**Background:**

The pre-hospital assessment non-specific complaint (NSC) often applies to patients whose diagnosis does not match any other specific assessment correlating to particular symptoms or diseases, though some previous studies have found them to be related to serious underlying conditions. The aim was to identify whether the structural factors such as urgency according to the dispatch priority of the Emergency Medical Communication Centre (EMCC) or work load in the Emergency Medical Services (EMS) are predisposing factors for the assessment of NSC instead of a specific assessment.

**Methods:**

All patients with assessed condition NSCs by the EMS to Södersjukhuset during 2011 (n = 493) were compared with gender- and age-matched controls (n = 493), which were randomly drawn from all patients with specific conditions in the EMS, regarding day of week, time of day and priority set by EMCC with chi-squared tests and multivariate logistic regression models.

**Results:**

Among patients with NSCs, more were females (58 %) and the median age was 82. Almost all patients were categorized with NSCs during the daytime (8 a.m. to 9 p.m.), i.e. 450 (91 %) as compared to 373 (75 %) of those with specific conditions (*p* < 0.01). The risk of having an EMS dispatched as low priority by the EMCC was almost doubled among patients with NSCs compared to controls (OR 1.97, 95 % CI 1.38–2.79).

**Conclusions:**

Since patients with NSCs appear most frequently during the hours with most transportations for the EMS, i.e. 10 a.m. to 2 p.m., and the risk of having the assessment NSC was doubled if the EMCC dispatched EMS as low priority, structural factors might be predisposing factors for the assessment.

## Background

The field of emergency medicine spans from the Emergency Medical Communication Center (EMCC) via the Emergency Medical Service (EMS) to the Emergency Department (ED), and timely provision of each link is an essential factor to secure a reliable healthcare system [1, 2]. To support the early symptom-based care in emergency medicine, management protocols for procedures and treatments are often available for specific complaints [3, 4]. However, every seventh patient admitted to EDs present with non-specific complaints (NSCs) [4, 5]. Patients with NSCs are challenging for EDs since their differential diagnoses range from lack of homecare to life-threatening conditions [4]. Furthermore, previous studies of patients presenting with NSCs have shown that these patients tend to be under-triaged, i.e. triaged to levels below that required by their actual medical condition, and (often suffer from serious conditions) and have high in-hospital mortality [6–8]. It is important to facilitate correct triage coordinated in the first two links, the EMCC and the EMS, as well as to minimize discrepancies between the first priority setting at the EMCC, at the scene by EMS and in the ED. In cases of over-triaging, a discrepancy may waste resources through unnecessary hospital transportation, have a negative impact on ambulance availability and cause ED crowding. In addition, under-triage may endanger patient safety and cause unfavorable outcomes [9, 10].

There is no specific definition of NSC [4], rather it is a subjective assessment by the EMS usually after ruling out other specific conditions in a patient with signs or symptoms that do not fit well into any other common assessment. Previous studies have demonstrated that patients with NSCs are at a high risk of suffering from an underlying serious condition. Also, patients described as decreased general condition [8], one of several non-specific presentations incorporated in NSCs, have a higher in-hospital mortality rate, especially in low triage priorities, than all other presenting complaints [4, 8]. This high risk of suffering a serious condition implies a lack of recognition of the seriousness of the condition at hand. Therefore, studying the chain of care in order to pinpoint changeable factors is important. Therefore, we hypothesized that the assessment of NSC was more common than assessments relating to specific conditions during periods of high work load for the EMS or when the EMCC indicates lower priority levels to the EMS. We have used the number of ambulance transportations per time unit as a proxy for work load and assumed that the higher demand for ambulance transports from the EMCC, given the fixed number of ambulances, the greater the pressure to shorten each ambulance mission, affecting the lower priority calls from EMCC the most.

## Method

### Study design

A registry based case-control study with prospectively collected data between 1 January 2011 and 31 December 2011 took place at Södersjukhuset, Stockholm, Sweden. Cases were defined as patients arriving with EMS to the ED at Södersjukhuset with NSCs in the EMS patient care record field “Assessed condition”. In total, 498 patients with NSCs were identified based on EMS records. Exclusion criteria were having left the ED without being seen by a physician (three patients) and being aged less than 18 years (two patients), leaving 493 patients in the study. After matching for age in exact years and sex, one control per case was randomly drawn from all patients arriving by EMS to the ED at Södersjukhuset during 2011, with all other assessment than NSC in the EMS patient care record fields “Assessed condition”.

### Settings

In Sweden there is one telephone number, 112, to call when in need of acute care or in the case of an emergency. The EMCC dispatchers are nurses, assistant nurses and people with other backgrounds. The EMCC follows the Swedish Medical Index to decide when to dispatch an EMS and can still simultaneously continue the call. Even some professionals needing an EMS for secondary transportations have to go through the EMCC. EMS is operated by two persons of which at least one is a nurse within the Stockholm area and use an own system were “Assessed condition” is set by the EMS personnel by selecting an appropriate code from a list of 140 different medical conditions that describe the patient’s symptoms. Södersjukhuset has a catchment area of approximately 500,000 people and has one of the largest EDs in Scandinavia with 115,000 visits each year. The Regional Ethical Review Board in Stockholm, Sweden approved the study.

### Data collection

Information about characteristics of the cases and controls including sex, age, day of week, time of the day as well as initial priority set by the EMCC and subsequently by the EMS, was collected through the electronic patient charts Akusys (version.5.0f, Södersjukhuset, Stockholm, Sweden, 1994), Melior (version 1.5, Siemens Medical Solutions, Siemens AB) and Pasett (It enheten Södersjukhuset, 1998-2008 version 1.61). Day of the week and time of day were extracted from the timestamp set when the EMCC decided to make the emergency call into a dispatch, i.e. not the time for when the EMS received the call or finished the call but the time EMCC sent for EMS. Dispatch priority was defined as the priority given by the EMCC using the Swedish Medical Index [11] when dispatching the EMS. EMS priority was, at the time of the study, not regulated; it was based on the experiential assessment of the patients’ condition and was four-tiered. Priority 1 corresponded to immediate life threat, priority 2; acute but not life-threatening, priority 3; transportation to hospital was required, while level 4 corresponded to no medical need during transport, and was not primarily performed by the ambulance service, but rather other transportation services. The EMS priority was defined as the final priority noted in the EMS report.

### Statistical analyses

Differences in characteristics between cases and controls were assessed with chi-square test at the significance level of 0.05. Gender was categorized into two groups (male and female) and age into 10 year intervals (18–29, 30–39, 40–49 and up to ≥90 years). Day of the week was categorized into weekday (Monday 07.00 to Friday 18.59) or weekend (Friday 19.00 to Monday 06.59) and time of day into day (08.00 to 20.59) or night (21.00 to 07.59), interval periods were selected based on clinical working shifts for EMS personnel. Furthermore, the time when the EMCC dispatched the EMS was plotted over 24 h for time of day. Both the EMCC and the EMS priority were categorized into high (priority 1 and priority 2) or low (priority 3 and priority 4). Differences in risk of presence of predisposing factors between cases and controls were assessed with multivariate logistic regression including time of day, EMCC priority and EMS priority in order to achieve odds ratios (ORs) with 95 % confidence intervals (95 % CIs). All analyses were performed with the statistical package STATA 10.0 for Windows (STATA Corp, College Station, TX).

## Results

### Characteristics of patients with non-specific complaints in the emergency medical service

Out of the 493 patients with NSCs in the EMS, 287 (58 %) were female (Table 1). The median age for all patients with NSC was 82 years. Due to matching for gender and age, results were similar in control group.

**Table 1:** Characteristics of patients with pre-hospital non-specific complaints (NSCs) and gender- and age-matched controls with specific pre-hospital assessments admitted to Södersjukhuset’s Emergency Department during 2012

	Pre-hospital NSC	Controls with specific pre-hospital assessment	
	Number (%)	Number (%)	*P*-value^€^
Total	493	493	
Sex	287 (58)	287 (58)	1.000
Female			
Age category			1.000
18–29	15 (3)	15 (3)	
30–39	9 (2)	9 (9)	
40–49	13 (3)	13 (3)	
50–59	17 (3)	17 (3)	
60–69	60 (12)	60 (12)	
70–79	91 (18)	91 (18)	
80–89	213 (43)	213 (43)	
≥90	75 (15)	75 (15)	
Day of the week			
Weekday	352 (71)	351 (71)	0.944
Weekend	141 (29)	142 (29)	
Time of the day			<0.001
Day	450 (91)	372 (75)	
Night	43 (9)	121 (24)	
Emergency Medical Communication Centre priority			<0.001
Low	182 (37)	86 (17)	
High	311 (63)	407 (83)	
Change in priority			
From low to high	5 (3*)	23 (27*)	<0.001
From high to low	106 (33**)	128 (31*)	0.30
Emergency Medical Service priority			<0.001
Low	286 (60)	191 (40)	
High	207 (41)	301 (59)	

^€^chi-square test

*Percentage = fraction of the patients dispatched with low priority by EMCC, i.e. 182 NSC and 86 controls

**Percentage = fraction of the patients dispatched with high priority by EMCC, i.e. 311 NSC and 407 controls

### Day of the week and time of day

**Fig. 1:**
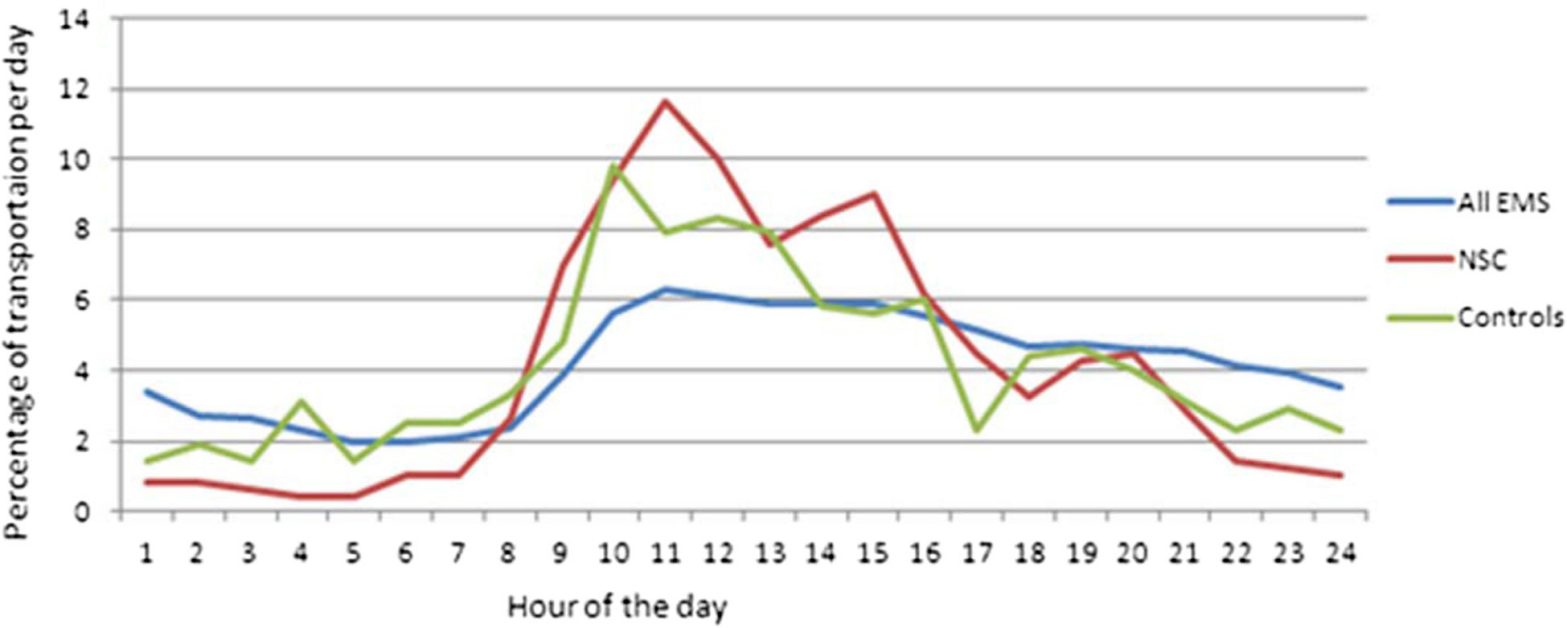
Time when the Emergency Medical Communication Centre dispatched the Emergency Medical Service (EMS) plotted over 24 h for time of day for 493 patients pre-hospital assessed by the EMS as a non-specific complaint (NSC) compared to 493 gender- and age-matched controls with specific assessments as well as all EMS transportation (40 600) admitted to Södersjukhuset’s Emergency Department during 2011

The majority of the patients received their assessment NSC on a weekday rather than a weekend (352 (71 %) and 141 (29 %), respectively); proportions were similar in controls (Table 1). 450 (91 %), of the patients received the assessment NSC in the daytime, compared with 325 (75 %) of the controls (*P*-value <0.001) (Table 1). The frequency of patients with NSCs peaked between approximately 10.00 a.m. and 2.00 p.m. while the frequency of patients with specific conditions had its peak at approximately 9 a.m. as well as the peak for all EMS transportations starts around 10.00 a.m (Fig. 1). Overall, there was a threefold increased risk of receiving the assessment of NSC in the daytime, (OR 3.47 95 % CI 2.37–5.08) compared with receiving a specific condition, according to the EMS assessment (Table 2).

**Table 2:** Association between structural factors and the pre-hospital assessment of non-specific complaint (NSC) compared to a gender- and age-matched control group with specific assessments admitted to Södersjukhuset’s Emergency Department during 2012

	Pre-hospital assessment NSC number (%) N = 493	Pre-hospital specific assessment Number (%) N = 493	Univariate odds ratio (95 % CI)	Multivariate odds ratio (95 % CI)^a^
Time of the day				
Day	450 (91)	372 (75)	3.56 (2.45–5.17)	3.47 (2.37–5.08)
Emergency Medical Communication Centre priority				
Low	182 (37)	86 (17)	2.78 (2.07–3.74)	1.97 (1.38–2.79)
Emergency Medical Service priority				
Low	284 (58)	192 (39)	2.18 (1.69–2.82)	1.66 (1.23–2.25)

^a^Multivariable logistic regression model including time of day, Emergency Medical Communication Centre priority and Emergency Medical Service priority

### Priority

The EMCC dispatched the EMS with a low priority in 182 (37 %) patients with NSCs as compared to 86 (17 %) with specific conditions, i.e. the controls (*p*-value <0.001) (Table 1). The odds of receiving the assessment NSC by the EMS was 1.97, (95 % CI 1.38–2.79) when the EMS was dispatched with a low priority by the EMCC (Table 2). The priority was changed from a low to a high priority by the EMS for 5 (2 %) patients with NSCs compared to for 23 (27 %) controls (*p*-value 0.007) among patients initially dispatched by the EMCC with a low priority (Table 1). The opposite, i.e. a change from high to low priority occurred in 102 (33 %) of the patients with NSC compared to 128 (31 %) of the controls (*p*-value **0.30**) (Table 1). The EMS priority noted in the EMS record were low in 284 (58 %) of patients with NSC compared to 192 (39 %) among controls (*p*-value <0.001) (Table 1).

## Discussion

This case control study confirmed the hypothesis that the NSCs was more common than specific assessments during hours with high work load, measured as the most number of transportations, i.e. approximately between 10 a.m. and 2 p.m., and when the EMS was dispatched with low priority from the EMCC. Furthermore, the EMCC priority is rarely changed by the EMS crew to higher priority among patients categorized as NSC, and therefore remains low in the EMS in the majority of patients.

The finding of an equal use of the assessment NSC between weekdays and weekends can be explained by the prerequisite for pre-hospital care, i.e. pre-hospital care has only minor weekend differences in requirements of level of competence, equipment or number of personnel involved in each of the EMCC calls or the EMS order. In comparison, previous studies showing a weekend effect have been performed in the in-hospital setting where diagnostic techniques and competence such as presence of professions and supervisors differs over the week [12].

In the 24-hour plot of the time the EMCC dispatched the EMS for the group with NSCs compared to specific conditions, there was an increase in patients at 10.00 a.m. and 02.00 p.m.; both time points overlap with the times with the highest frequency of patients transported by the EMS to EDs in Stockholm, Sweden. This finding, i.e. a more common use of the assessment NSC during the busiest hours, can be speculated to be a result of the workload (as described in the background) and the EMS crews performing a minimal number of procedures to speed up the process. Another reason for NSCs appearing during daytime might be that patients with less clinical urgency may visit an ED after they have been denied a visit to primary care [13] or even admitted to hospital for non-medical reasons, i.e. social needs or admission for long-term care instead of an acute condition in need of an ED’s resources [14].

Regarding priority, the assessment of NSC was more common when the EMS was dispatched with a low priority by the EMCC. When an EMS is dispatched with low priority to patients with unknown problems based on the information given to the EMCC during communication, it has generally been shown to be difficult to dispatch the EMS correctly [15, 16]. In the current study, the priority set by the EMCC was more often changed by EMS crew, both to higher and lower, in patients with specific assessments than those with NSCs. A reason to change dispatch priority is that the EMS personnel finds the patient’s needs less urgent than assessed by the EMCC (16). However, one has to bear in mind that changing priority does not necessarily reflect poorly on EMCC as triage since the settings for EMCC and EMS differ, i.e. setting priority in a non-visual environment compared to face-to face with the patient and possibilities to both assess vital signs and start treatment. Moreover, the objective for the EMCC is to promote patient safe priorities and if in doubt to err to the higher priority level.

The larger portion with low priority in the EMS compared to the portion with low priority set by the EMCC is determined by with the findings of other studies. Hjälte et al. (16) found that when a disagreement in level of priority was present between the dispatch center and EMS personnel, it was commonly the case that the EMS personnel assigned a lower priority than the dispatcher.

Weaknesses of the study include the lack of an agreed definition of the assessment of NSC, lack of information about what conditions were excluded before NSC was set as the main assessment, and the assessment and priority system has not been validated. Also, the reason why the EMS personnel did the down-prioritizing in our study was unfortunately not recorded in the EMS patient care record system. However, one has to bear in mind that changing priority does not necessarily reflect poorly on EMCC as triage since the settings for EMCC and EMS differ, i.e. setting priority in a non-visual environment compared to face-to face with the patient and possibilities to both assess vital signs and start treatment which reduces the urgency. In addition a mission aim of the EMCC is to guarantee patient safely, which will lead to the strategy of erring to the side of over triage rather than the opposite.

Further, the study’s design itself comes with some limitations, even if we matched for gender and age our design with a one-to-one ratio and lack of a priori knowledge about underlying conditions might have rendered a non-representative control group. Finally, the classification of priority to be a structural factor is not clear cut since it also might be classified as a patient-related factor, i.e. the priority is based on the patient and his or her history and vital signs. However, since priority is set on similar basis for all patients, i.e. not adjusted for age, co-morbidity or even drugs affecting vital signs such as betablockers it might still be classified as a structural factor. Further, it is also a structural factor in the sentence that the amount of NSC seems to be comparable over time and it is set based on variables selected due to our current knowledge and believing.

Methodological strengths of the study include the design as a large pragmatic study over one calendar year using registries for data collection and the use of an age- and gender-matched control group.

## Conclusions

These results indicate that assessment of NSCs in the pre-hospital settings is associated with structural factors such as work lead, measured as times of the most number of transportations in the daytime and low priority by the EMCC and EMS. Future studies need to focus on in-depth reasons for the use of NSC by EMS as well as to distinguish the difference between those with an urgent need of care among the patients with NSC from others that do not need immediate medical attention.
